# Oxytocin Regulates Synaptic Transmission in the Sensory Cortices in a Developmentally Dynamic Manner

**DOI:** 10.3389/fncel.2021.673439

**Published:** 2021-06-09

**Authors:** Jing Zhang, Shu-Jing Li, Wanying Miao, Xiaodi Zhang, Jing-Jing Zheng, Chen Wang, Xiang Yu

**Affiliations:** ^1^Institute of Neuroscience, State Key Laboratory of Neuroscience, Center for Excellence in Brain Science and Intelligence Technology, Chinese Academy of Sciences, Shanghai, China; ^2^Department of Clinical Pharmacology, Pharmacy College, Nanjing Medical University, Nanjing, China; ^3^CAS Key Laboratory of Biological Effects of Nanomaterials and Nanosafety, CAS Center for Excellence in Nanoscience, National Center for Nanoscience and Technology, Beijing, China; ^4^School of Life Sciences, Peking-Tsinghua Center for Life Sciences, and Peking University McGovern Institute, Peking University, Beijing, China

**Keywords:** oxytocin, oxytocin receptor, synaptic transmission, pyramidal neurons, primary somatosensory cortex, critical period, sensitive period

## Abstract

The development and stabilization of neuronal circuits are critical to proper brain function. Synapses are the building blocks of neural circuits. Here we examine the effects of the neuropeptide oxytocin on synaptic transmission in L2/3 pyramidal neurons of the barrel field of the primary somatosensory cortex (S1BF). We find that perfusion of oxytocin onto acute brain slices significantly increases the frequency of miniature excitatory postsynaptic currents (mEPSC) of S1BF L2/3 pyramidal neurons at P10 and P14, but reduces it at the later ages of P22 and P28; the transition occurs at around P18. Since oxytocin expression is itself regulated by sensory experience, we also examine whether the effects of oxytocin on excitatory synaptic transmission correlate with that of sensory experience. We find that, indeed, the effects of sensory experience and oxytocin on excitatory synaptic transmission of L2/3 pyramidal neurons both peak at around P14 and plateau around P18, suggesting that they regulate a specific form of synaptic plasticity in L2/3 pyramidal neurons, with a sensitive/critical period ending around P18. Consistently, oxytocin receptor (*Oxtr*) expression in glutamatergic neurons of the upper layers of the cerebral cortex peaks around P14. By P28, however, *Oxtr* expression becomes more prominent in GABAergic neurons, especially somatostatin (SST) neurons. At P28, oxytocin perfusion increases inhibitory synaptic transmission and reduces excitatory synaptic transmission, effects that result in a net reduction of neuronal excitation, in contrast to increased excitation at P14. Using oxytocin knockout mice and *Oxtr* conditional knockout mice, we show that loss-of-function of oxytocin affects baseline excitatory synaptic transmission, while *Oxtr* is required for oxytocin-induced changes in excitatory synaptic transmission, at both P14 and P28. Together, these results demonstrate that oxytocin has complex and dynamic functions in regulating synaptic transmission in cortical L2/3 pyramidal neurons. These findings add to existing knowledge of the function of oxytocin in regulating neural circuit development and plasticity.

## Introduction

The wiring of neural circuits is an intricate developmental process, regulated by a combination of intrinsic and extrinsic cues (Katz and Shatz, 1996; Crair, 1999; Sur and Rubenstein, 2005; Blankenship and Feller, 2010). In rodents, wiring of the cerebral cortex occurs mostly during the first 4 weeks of postnatal development (Micheva and Beaulieu, 1996). This process is regulated by genetic programming, in combination with environmental factors (Feldman and Brecht, 2005; Fox and Wong, 2005; Nithianantharajah and Hannan, 2006; Sale et al., 2009). The anatomical and functional properties of neurons in the sensory cortices are particularly sensitive to modification by environmental stimuli during a limited developmental window, known as the “sensitive period” (Knudsen, 2004; Luby et al., 2020). An extreme form of sensitive period is the “critical period”, where appropriate experience is essential for the normal development of a pathway or set of connections (Hensch, 2004). The most well-studied example of the critical period is the formation of ocular dominance columns in the visual cortex (Wiesel and Hubel, 1963). Subsequent studies showed that different aspects of visual cortical development have different sensitive/critical periods (Hensch, 2004; Hooks and Chen, 2007). Other cortical regions also have various sensitive/critical periods for different aspects of their development (Neville and Bavelier, 2002; Erzurumlu and Gaspar, 2012; Kral, 2013).

In previous work, we identified a new form of experience-dependent cross-modal plasticity in the sensory cortices, by showing that deprivation of sensory inputs in one modality cross-modally delayed the development of other sensory cortices (Zheng et al., 2014). Specifically, we showed that deprivation of somatosensory inputs through whisker deprivation (WD) reduced excitatory synaptic transmission in L2/3 pyramidal neurons of both the barrel field of the primary somatosensory cortex (S1BF), and the primary visual cortex (V1), at both P7 and P14. We further showed that the neuropeptide oxytocin, mostly synthesized in the paraventricular nuclei of the hypothalamus (PVH) and the supraoptic nuclei (SON), is an important mediator of this form of plasticity. Specifically, at P14, oxytocin knockout mice had reduced excitatory synaptic transmission, similar to the effects of WD, while perfusion of oxytocin onto acute brain slices or *in vivo* injection of oxytocin enhanced excitatory synaptic transmission (Zheng et al., 2014).

An important remaining question is whether this form of experience- and oxytocin-dependent plasticity has a sensitive/critical period. Here, we address this question by examining the effect of experience and oxytocin on synaptic transmission on L2/3 pyramidal neurons at different developmental time points. Our results show that this experience-dependent plasticity in the sensory cortices has a critical period ending around P18. We further show that the effect of oxytocin on excitatory and inhibitory synaptic transmission, as well as the expression of oxytocin receptors in the cerebral cortex, change during cortical development. Together, these results demonstrate that the developmental effects of oxytocin on excitatory and inhibitory synaptic transmission are dynamic and complex.

## Materials and Methods

### Animals

All animal procedures complied with the animal care standards set forth by the US National Institutes of Health and were approved by the Institutional Animal Care and Use Committee at the Institute of Neuroscience, Chinese Academy of Sciences, and of Peking University. Mice on C57BL/6 background were raised in a specific pathogen-free (SPF) environment and group-housed under a 12h–12 h light-dark cycle with food and water provided *ad libitum* from the cage lid. Their health status was monitored routinely.

**Figure 1:**
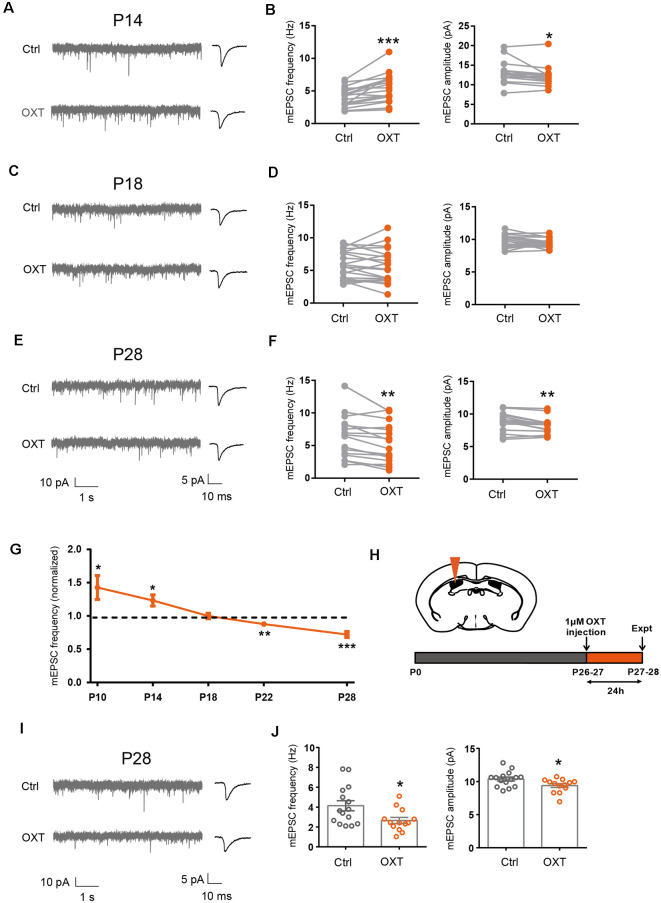
Dynamic regulation of excitatory synaptic transmission by oxytocin. **(A,C,E)** Representative mEPSC recordings (left) and average waveforms (right) from S1BF L2/3 pyramidal neurons before (Ctrl) and after oxytocin (OXT) application, age as indicated. **(B)** Oxytocin application increased mEPSC frequency but reduced mEPSC amplitude in P14 mice (frequency: Ctrl, 4.10 ± 0.35 Hz; OXT, 5.43 ± 0.52 Hz; *n* = 18; *P* < 0.001, paired *t*-test; amplitude: Ctrl, 13.01 ± 0.63 pA; OXT, 12.17 ± 0.58 pA; *P* < 0.05, paired *t*-test). **(D)** Oxytocin application did not significantly affect mEPSC frequency or amplitude at P18 (frequency: Ctrl, 5.80 ± 0.47 Hz; OXT, 5.65 ± 0.57 Hz; *n* = 21; *P* = 0.60, paired *t*-test; amplitude: Ctrl, 9.71 ± 0.21 pA; OXT, 9.39 ± 0.15 pA; *P* = 0.08, paired *t*-test). **(F)** Oxytocin application reduced both mEPSC frequency and amplitude in P28 mice (frequency: Ctrl, 6.35 ± 0.82 Hz; OXT, 5.40 ± 0.76 Hz; *n* = 16; *P* < 0.01, paired *t-*test; amplitude: Ctrl, 8.75 ± 0.36 pA; OXT, 8.24 ± 0.31 pA; *P* < 0.01, paired *t*-test). **(G)** Developmental effects of oxytocin on mEPSC frequency (effect of oxytocin application normalized to before application control; P10: 1.43 ± 0.18; *n* = 10; *P* < 0.05; P14: 1.23 ± 0.08; *n* = 9; *P* < 0.05 ; P18: 0.96 ± 0.03; *n* = 12; *P* = 0.21; P22: 0.88 ± 0.04; *n* = 16; *P* < 0.01; P28: 0.72 ± 0.05; *n* = 12; *P* < 0.001, paired *t-*test at each time point). **(H)** Schematic of experimental procedure for *in vivo* oxytocin injection. **(I)** Representative mEPSC recordings (left) and average waveforms (right) for conditions as indicated in P28 mice. **(J)** mEPSC frequency and amplitude 24 h following *in vivo* oxytocin injection in P28 mice (frequency: Ctrl, 4.14 ± 0.50 Hz, *n* = 15; OXT, 2.66 ± 0.31 Hz, *n* = 13; *P* < 0.05, unpaired *t*-test; amplitude: Ctrl, 10.38 ± 0.30 pA; OXT, 9.40 ± 0.29 pA; *P* < 0.05, unpaired *t*-test). In this and subsequent figures, error bars represent SEM; *P < 0.05, **P < 0.01, ***P < 0.001.

*GAD67*-*GFP* mice [also known as Gad1^tm1.1Tama^; GAD67-GFP (delta neo)] (Tamamaki et al., 2003) were gifts of Yuchio Yanagawa (Department of Genetic and Behavioral Neuroscience, Gunma University Graduate School of Medicine, Maebashi, Japan). The* Oxt*^+/–^ mice (*B6; 129S-Oxt^tm1Wsy^/J*; JAX: 002713, RRID: IMSR_JAX:002713) on C57/BL6 background (Young et al., 1996) were gifts of Dr. Scott Young (US National Institute of Mental Health). *E2a-Cre* mice [B6.FVB-Tg (EIIa-cre) C5379Lmgd/J; JAX: 003724, RRID: IMSR_JAX:003724] express Cre recombinase in the early embryo, prior to implantation (Lakso et al., 1996). *Nex-Cre* mice (gift of Prof. Klaus Nave, Max Planck Institute, Goettingen, Germany) express Cre recombinase in excitatory neurons of the cerebral cortex and hippocampus starting from the late embryo (Goebbels et al., 2006). Oxytocin receptor conditional knockout mice (*Oxtr^flox/flox^*; full name: B6.129 (SJL)-Oxtr^tm1.1Wsy^/J; JAX:008471, RRID:IMSR_JAX:(008471) (Lee et al., 2008) were crossed with *E2a-Cre* or *Nex-Cre* to generate homozygous floxed and heterozygous Cre mice. Littermate homozygous floxed mice not expressing Cre were used as controls. The P7 group consists of P7–P9 mice, the P10 group consists of P9–P11 mice, the P14 group consists of P14–P15 mice, the P18 group consists of P17–P19 mice, the P21 group consists of P21–P22 mice, the P28 group consists of P26–P30 mice, and the adult group consists of 2–4 month-old mice. For data presented in Figure 1G, mice in the P18 and P22 groups were of exact ages. Both male and female mice were used for all experiments.

For whisker deprivation (WD) experiments, littermates were randomly assigned to the control or WD group. All pups underwent anesthesia (isoflurane), but only mice assigned to the WD group had their whiskers trimmed from P0–P3 and plucked every other day from P4 until the time of the experiment. For dark-rearing (DR) experiments, pregnant dams were randomly placed in a cage completely covered by thick black plastic 1–4 days before delivery, and mice were cared for under dim red light. Mice were dark-reared until the time of experiment; control mice were from litters raised in parallel, under standard lighting conditions.

### Acute Brain Slice Preparation

Brain slices from deeply anesthetized mice (0.14 g/kg sodium pentobarbital) were essentially prepared as previously described (Zheng et al., 2014). Brains were rapidly removed and immersed in ice-cold dissection solution. For young mice (P7–P21), choline-based dissection buffer contained (in mM): CholineCl 110, KCl 2.5, NaH_2_PO_4_ 1.3, MgCl_2_ 7, CaCl_2_ 0.5, NaHCO_3_ 25, glucose 20, bubbled with 95%O_2_/5% CO_2_, pH 7.4. Brains slices of adolescent mice (P21–P28) were cut in N-Methyl-D-glucamine (NMDG) based solution containing (in mM): NMDG 93, KCl 2.5, NaH_2_PO_4_ 1.2, MgSO_4_ 10, CaCl_2_ 0.5, NaHCO_3_ 30, HEPES 20, glucose 25, sodium pyruvate 3, titrated to pH 7.3–7.4 by adding approximately 8.5 ml of 10 M HCl to 1 liter of solution, and bubbled with 95%O_2_/5% CO_2_. Coronal slices were cut at 300–350 μm using a Vibratome 3000 (Leica, Germany) microslicer. Brain slices cut in choline solution were allowed to recover in a submersion holding chamber with artificial cerebral spinal fluid (aCSF) consisting of (in mM): NaCl 125, KCl 2.5, NaH_2_PO_4_ 1.3, MgCl_2_ 1.3, CaCl_2_ 2, NaHCO_3_ 25, glucose 20, bubbled with 95%O_2_/5% CO_2_ for 30 min at 37°C and a further 60 min at 25–28°C prior to recordings. Brain slices cut in NMDG allowed to recover in a submersion holding chamber with bubbled with 95%O_2_/5% CO_2_NMDG-based dissection buffer for 7 min, followed by a further 60 min in aCSF at 25–28°C prior to recordings. Brain slices of P21 mice cut with choline or NMDG-based solutions yielded similar results and were pooled.

### Whole-Cell Recordings

Whole-cell recordings of L2/3 pyramidal neurons were made with a MultiClamp 700B amplifier (Molecular Devices, Sunnyvale, CA, USA), as previously described (Zheng et al., 2014). Signals were low-pass filtered at 2 kHz and digitized at 10 kHz using Digidata 1332A (Molecular Devices). Cells were visualized with an upright microscope (Nikon FN1, Japan) and a 40x water immersion objective under infrared optics. Slices were perfused with oxygenated aCSF at a rate of 4–6 ml/min at 28–30°C and used within 6 h of the first recording. S1BF and V1 were identified according to standard stereotaxic coordinates. For mEPSC recordings, Cs^+^-based internal solution containing CsMeSO_4_ 130, CsCl 5, HEPES 10, EGTA 0.5, Na_2_ATP 15, MgATP 4 and Na_3_GTP 0.3 and sodium phosphocreatine 10 (pH 7.4, 270–280 mOsm) was used. For mIPSC recordings, high chloride Cs^+^-based internal solution containing (in mM): CsCl 110, NaCl 10, MgCl_2_ 5, EGTA 0.5, MgATP 2, Na_3_GTP 0.3 and HEPES 40 (pH 7.4, 270–280 mOsm) was used.

Recordings were made from 2–3 cells per slice, and 2–3 slices per mouse; for drug bath application experiments, one cell per slice was recorded. For mEPSC recordings, cells were held at −70 mV in voltage-clamp, with pipette resistance of 3–4 MΩ in the presence of tetrodotoxin (TTX, 0.5 μM) and picrotoxin (50 μM) to block Na^+^ channels and GABA_A_R, respectively. For mIPSC recordings, cells were held at −60 mV in the presence of TTX (0.5 μM) and NBQX (10 μM) to block Na^+^ channels and AMPAR respectively. A brief hyperpolarization (10 mV, 100 ms) was given to monitor series and input resistances every 10 s. Cells with changes of input or series resistance greater than 20% were excluded from analyses. All cells analyzed had a series resistance <25 MΩ. Liquid junction potential and series resistance were uncompensated. Data were analyzed in MiniAnalysis (Synaptosoft, Fort Lee, NJ) with detection thresholds of 5 pA and 6 pA, for mEPSC and mIPSC, respectively. Data were analyzed blinded to the experimental condition.

All salts and drugs were obtained from Sigma or Tocris, except for TTX obtained from the Hebei Fisheries Science and Technology Development Company (Qinhuangdao, Hebei Province, China), and oxytocin from Guoping Pharmaceutical (Hefei, Anhui Province, China). *In vivo* stereotaxic injections were performed as previously described (Zheng et al., 2014). Oxytocin (1 μM, 3 μl, unilateral) was injected into the lateral ventricle (bregma: −0.3 mm; lateral: 1.3 mm; ventral: 1.7 mm). FITC-oxytocin (CYIQNCPLG[DD-miniPEG]-K(FITC)-NH2, 1 μM, 1 μl, unilateral) was injected into to the PVH (bregma: −0.7 mm; lateral: 1.7 mm; ventral: 4.0 mm; offset angel: 7°C) using a stereotaxic instrument (RWD Life Science, Shenzhen, China) and a syringe pump (Harvard Apparatus), at a speed of 0.2 μl/min. Sections were cut 24 h after injection and recordings were made from the injected side.

### Fluorescent* In situ* Hybridization, Immunohistochemistry, and Quantitation

*In situ* hybridization was performed as previously described (Wu et al., 2009; Xiu et al., 2014; Duan et al., 2018). *Oxtr* probes were cloned into the BamHI and EcoRI sites of pBluescript vector using forward primer cgcggatccGTTGGCACGGGTCAGTAGT, and reverse primer ccggaattcAATGCTTTCTGGGATGTCCTAA. RNA probes were labeled using DIG RNA Labeling Mix (Roche, Cat# 11277073910). Anti-Digoxigenin-AP Fab fragments (Roche, Cat# 11093274910, RRID: AB_514497) were used for DIG labeling of *Oxtr*. For colocalization with various cell type markers, the following primary antibodies were co-incubated with anti-digoxigenin-AP: CaMKIIβ (Abcam, Cat# ab34703, RRID: AB_2275072) and Somatostatin (SST, Santa Cruz, Cat# sc-7819, RRID: AB_2302603). The following secondary antibodies were used: Donkey anti-Rabbit Alexa Fluor 488 (Thermo Fisher Scientific, Cat# A-21206, RRID: AB_2535792), Donkey anti-Goat Alexa Fluor 488 (Thermo Fisher Scientific, Cat# A-11055, RRID: AB_2534102), both diluted 1:1,000. Fast red (Roche, Cat# 11496549001) was used for visualization of *in situ* hybridization. GAD67-GFP transgenic mice were used to label GABAergic neurons. Sections were mounted with Fluoromount medium (Sigma–Aldrich, Cat# F4680).

S1 Layer 2/3 images (1,024 × 1,024) were acquired on a Nikon A1 confocal microscope with S Fluor 40× Oil DIC H N2 Optics (N.A. = 1.3). Image analysis was performed using Image-Pro Plus (Media Cybernetics, Rockville, MD, USA), blinded to the experimental condition. For colocalization analysis, images of each channel were separately thresholded, and colocalization was defined as one or more pixels of overlap between the two conditions. *Oxtr* in each cell type was measured as a ratio of total *Oxtr* area in the section. For measuring the percentage of *Oxtr* positive marker, the area of *Oxtr* colocalizing with each marker was ratioed over that of total marker area. Image analysis was carried out with no post-acquisition modifications. For example images, brightness/contrast was adjusted with linear ranges using ImageJ (N.I.H., Bethesda, MD, USA). P14 and P28 sections were adjusted with the same parameters.

### Oxytocin Binding in N2a Cells

Cultured N2a cells were transfected with pCS2 or pCS2-HAmOXTR. Live cells were then incubated with aCSF containing 1 mM oxytocin (GL Biochem) for 20 min, and then fixed in 4% PFA for 1 h. As control, cells were incubated in aCSF containing 1 mM Vasopressin (GL Biochem). Immunostaining was performed with the following antibodies: Oxytocin (Phoenix Pharmaceuticals, Cat# G-051–01, RRID: AB_2876858; 1:250), HA (Covance, Cat# MMS-101P, RRID: AB_2314672; 1:200), Donkey anti-rabbit Alexa Fluor 488 (Thermo Fisher Scientific, Cat# A-21206, RRID:AB_2535792; 1:500) and Donkey anti-mouse Alexa Fluor 568 (Thermo Fisher Scientific, Cat# A10037; 1:500). For the antibody block experiment, the oxytocin antibody was pre-incubated with 10 mM oxytocin. Sections were incubated with 640/660 Deep-Red Fluorescent Nissl Stain (Thermo Fisher Scientific Cat# N21483) for 20 min at room temperature, washed, and mounted onto glass slides in 70% glycerol. Images were acquired on a Nikon A1 confocal microscope with a Plan Apo VC 20× DIC N2 (N.A. = 0.8) objective.

### Oxytocin Binding in Acute Brain Slices

To visualize the ability of cells to bind to oxytocin, acute brain slices (sectioned as in “Acute Brain Slice Preparation”) were incubated in aCSF containing 1 mM oxytocin (GL Biochem) or 1 mM FITC-oxytocin (Guoping Pharmaceutical, Hefei, Anhui Province, China) for 20 min, then fixed in 4% PFA overnight. As control, brain slices were incubated in aCSF containing 1 mM Vasopressin (GL Biochem). Immunostaining was performed the following day after brain sections were washed in PBS. The following antibodies were used: Oxytocin (Phoenix Pharmaceuticals, Cat# G-051–01, RRID: AB_2876858; 1:250), NeuN (Millopore Cat# MAB377, RRID: AB_2298772; 1:250), Donkey anti-rabbit Alexa Fluor 568 (Thermo Fisher Scientific, Cat# A10042 RRID: AB_2534017; 1:500) and Donkey anti-mouse Alexa Fluor 647 (Thermo Fisher Scientific, Cat# A-31571, RRID: AB_162542; 1:500). Sections were incubated with DAPI (Thermo Fisher Scientific Cat# D1306 RRID: AB_2629482) for 15 min at room temperature, washed and mounted onto glass slides in 70% glycerol. Images were acquired on a Nikon A1 confocal microscope with a Plan Apo 10× DIC L (N.A. = 0.45) or a Plan Apo VC 20× DIC N2 (N.A. = 0.75) objective.

Perfusion of P14 S1BF brain slices with FITC-oxytocin (1 μM) significantly increased mEPSC frequency (Ctrl: 5.10 ± 0.27 Hz, FITC-OXT: 6.66 ± 0.39 Hz; *P* < 0.001) and reduced mEPSC amplitude (Ctrl: 12.00 ± 0.29 pA, OXT: 11.39 ± 0.32 pA; *P* < 0.05), similar to the effects of untagged oxytocin (Figures 1A,B), suggesting that the FITC tag did not significantly interfere with the function of oxytocin in regulating excitatory synaptic transmission.

### Real-Time qPCR and Oxytocin Peptide Measurements

Total mRNA was extracted from the cerebral cortex, hippocampus, and hypothalamus, using TRIzol reagent (Invitrogen, Cat# 15596018). First-strand cDNA was generated using the M-MLV reverse transcriptase (Promega, Cat# M1701) according to the manufacturer’s protocols. Real-time qPCR was performed using SYBR Green Master Mix (TaKaRa, Cat# RR420A) on a LightCycler 480 (Roche Applied Science). All reactions were carried out in duplicates, and the comparative C_T_ method was used for comparisons between samples. The following primers were used: *Oxtr-*1-F CCGCACAGTGAAGATGACCT; *Oxtr*-1-R AGCATGGCAATGATGAAGGCAG; *Gapdh*-F CTGCCCAGAACATCATCCCT; *Gapdh*-R TGAAGTCGCAGGAGACAACC.

Oxytocin peptide concentration from S1, V1 and plasma were measured using an ELISA kit (Pheonix Pharmaceutics, EK-051–01), as previously described (Zheng et al., 2014).

### Statistical Analysis

Statistical analysis was performed using GraphPad Prism 7 (GraphPad Software, La Jolla, CA, USA). Data are presented as mean ± SEM. Unpaired two-tailed Student’s *t*-test (for sample pairs) or one-way ANOVA (for three or more samples) followed by Tukey’s multiple comparison tests were used, depending on the number of samples. For oxytocin perfusion experiments, paired two-tailed Student’s *t*-test were used. For electrophysiological experiments, *n* represents the number of neurons. For *in situ* hybridization experiments, *n* represents the number of brain sections. For other experiments, *n* represents the number of mice. Typically, three or more mice were used for each experimental condition. Data were analyzed blinded to the experimental condition.

## Results

### Experience- and Oxytocin-Dependent Regulation of Excitatory Synaptic Transmission Has a Sensitive/Critical Period

In previous work, we showed that whisker deprivation (WD) from birth (see “Materials and Methods” section) significantly reduced the frequency of miniature excitatory postsynaptic currents (mEPSC) of L2/3 pyramidal neurons in both S1BF and V1, at both P7 and P14 (Zheng et al., 2014). L2/3 pyramidal neurons of the cerebral cortex receive excitatory synaptic inputs from L4 ascending axons, as well as from other L2/3 neurons (Petersen, 2007), and thus functioning as important integrators of inputting sensory information. Here recording from L2/3 neurons of P18 mice, we found no significant differences in mEPSC frequency between WD mice and their whisker intact littermates (Supplementary Figures 1A,B), in both S1BF (Ctrl: 1.95 ± 0.34 Hz; WD: 1.90 ± 0.30 Hz; *P* = 0.93) and V1 (Ctrl: 1.81 ± 0.25 Hz; WD: 1.91 ± 0.29 Hz; *P* = 0.78).

Following dark-rearing (DR), another unimodal sensory deprivation paradigm, similar results were obtained. DR litters showed a significant reduction in mEPSC frequency at the earlier time points of P7 and P14 (Zheng et al., 2014); at P18, however, mEPSC frequency was not different between DR mice and those reared under standard lighting conditions (Ctrl; Supplementary Figures 1C,D), in both S1BF (Ctrl: 1.71 ± 0.27 Hz; DR: 1.52 ± 0.23 Hz; *P* = 0.62) and V1 (Ctrl: 1.83 ± 0.40 Hz; WD: 1.56 ± 0.28 Hz; *P* = 0.57). Together, these results suggest that experience-dependent synaptic plasticity in the sensory cortices has a sensitive/critical period ending around P18.

Since we have previously shown that sensory experience regulated excitatory synaptic transmission *via* the neuropeptide oxytocin (Zheng et al., 2014), we asked if oxytocin regulated excitatory synaptic transmission with a similar sensitive/critical period. We thus bath applied oxytocin (1 μM) onto acute S1BF brain slices of P14, P18, and P28 mice, and measured mEPSC frequency and amplitude of S1BF L2/3 pyramidal neurons, before and after oxytocin application. Consistent with our previous report (Zheng et al., 2014), in P14 mice, oxytocin significantly increased mEPSC frequency (Ctrl: 4.10 ± 0.35 Hz, OXT: 5.43 ± 0.52 Hz; *P* < 0.001; Figures 1A,B). We also observed a small, but significant, reduction in mEPSC amplitude (Ctrl: 13.01 ± 0.63 pA, OXT: 12.17 ± 0.58 pA; *P* < 0.05; please see “Discussion” section for discussion on all mEPSC amplitude changes). mEPSC frequency, reflecting release probability of individual synapses and total synapse number of the cell, and mEPSC amplitude, reflecting the size of individual synapses, both contribute to total synaptic strength. To more directly measure the effect of oxytocin on the total synaptic inputs of L2/3 neurons, we calculated the total charge transfer per second and found it to be significantly higher following oxytocin application (Ctrl: 148.32 ± 11.23 pAms; OXT: 193.10 ± 16.35 pAms; *P* < 0.01), consistent with oxytocin increasing total excitatory synaptic input of L2/3 pyramidal neurons at this developmental stage.

In P18 mice, oxytocin application had no significant effects on mEPSC frequency (Ctrl: 5.80 ± 0.47 Hz, OXT: 5.65 ± 0.57 Hz; *P* = 0.60) or amplitude (Ctrl: 9.71 ± 0.21 pA, OXT: 9.39 ± 0.15 pA; *P* = 0.08; Figures 1C,D). This result suggested that the effect of oxytocin on promoting excitatory synaptic transmission also had a critical period ending around P18, similar to the sensitive/critical period observed following sensory deprivation by WD or DR (Supplementary Figures 1A–D). Consistently, at P18-P21, sensory deprivation by WD or DR did not significantly reduce oxytocin peptide level, in S1 or V1 (Supplementary Figures 1E,F). This contrasts with significantly reduced oxytocin levels in S1 and V1 of P14 mice following WD or DR (Zheng et al., 2014).

At P28, oxytocin perfusion significantly reduced mEPSC frequency (Ctrl: 6.35 ± 085 Hz, OXT: 5.40 ± 0.76 Hz; *P* < 0.01) and amplitude (Ctrl: 8.75 ± 0.36 pA, OXT: 8.24 ± 0.31 pA; *P* < 0.01; Figures 1E,F).

To confirm the above results, we performed an additional set of experiments, with five time points between P10 to P28. At the earlier time points of P10 and P14, oxytocin significantly increased mEPSC frequency, while at the later time points of P22 and P28, it significantly reduced it; at the P18 transition point, oxytocin did not significantly affect excitatory synaptic transmission (all results shown as fold changes following oxytocin application, normalized to before application value; P10: 1.43 ± 0.18, *P* < 0.05; P14: 1.23 ± 0.08; *P* < 0.05; P 18: 0.96 ± 0.03; *P* = 0.21; P22: 0.88 ± 0.04, *P* < 0.01; P28: 0.72 ± 0.05, *P* < 0.001; Figure 1G).

In previous work, we showed that *in vivo* administration of oxytocin into the S1 of P12/13 mice significantly increased mEPSC frequency of L2/3 pyramidal neurons measured 24 h later (Zheng et al., 2014). We performed the same assay in P26/27 mice (Figure 1H), by injecting oxytocin into the lateral ventricle and recording from L2/3 pyramidal neurons 24 h later. We found significant reduction in mEPSC frequency (Ctrl: 4.14 ± 0.50 Hz, OXT: 2.66 ± 0.31 Hz; *P* < 0.05) and amplitude (Ctrl: 10.38 ± 0.30 pA, OXT: 9.40 ± 0.29 pA; *P* < 0.05) in mice injected with oxytocin, as compared to control mice injected with saline (Figures 1I,J).

Together, the above results show that regulation of excitatory synaptic transmission in L2/3 neurons by experience and oxytocin has sensitive/critical periods ending around P18. Furthermore, oxytocin regulates excitatory synaptic transmission with a dynamic time course, increasing excitatory synaptic strength at P14 and reducing it at the later time point of P28.

### Developmental Effects of Oxytocin on Inhibitory Synaptic Transmission

**Figure 2:**
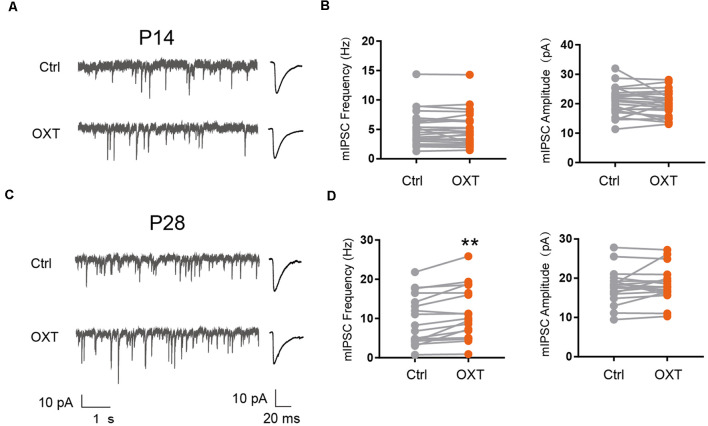
Developmental effects of oxytocin application on inhibitory synaptic transmission. **(A–C)** Representative mIPSC recordings (left) and average waveforms (right) from S1BF L2/3 pyramidal neurons before (Ctrl) and after oxytocin (OXT) application, age as indicated. **(B)** Oxytocin application did not significantly affect mIPSC frequency or amplitude in P14 mice (frequency: Ctrl, 5.02 ± 0.58 Hz; OXT, 5.0 ± 0.58 Hz; *n* = 25; *P* = 0.87, paired *t*-test; amplitude: Ctrl, 20.82 ± 0.94 pA; OXT, 20.17 ± 0.85 pA; *P* = 0.31, paired *t*-test). **(D)** Oxytocin application increased mIPSC frequency but did not affect mIPSC amplitude in P28 mice (frequency: Ctrl, 9.17 ± 1.41 Hz; OXT, 10.77 ± 1.53 Hz; *n* = 19; **P < 0.01, paired *t*-test; amplitude: Ctrl, 17.82 ± 0.99 pA; OXT, 18.38 ± 0.99 pA; *P* = 0.33, paired *t*-test). **P < 0.01.

We next examined the effect of oxytocin on inhibitory synaptic transmission, by measuring the frequency and amplitude of miniature inhibitory post-synaptic currents (mIPSC). At P14, oxytocin application did not significantly affect mIPSC amplitude (Ctrl: 20.82 ± 0.94 pA, OXT: 20.17 ± 0.85 pA; *P* = 0.31) or frequency (Ctrl: 5.02 ± 0.58 Hz, OXT: 5.0 ± 0.58 Hz; *P* = 0.87; Figures 2A,B). At P28, however, oxytocin application significantly increased mIPSC frequency (Ctrl: 9.17 ± 1.41 Hz, OXT: 10.77 ± 1.53 Hz; *P* < 0.01), but did not affect mIPSC amplitude (Ctrl: 17.82 ± 0.99 pA, OXT: 18.38 ± 0.99 pA; *P* = 0.33; Figures 2C,D). Thus, the effect of oxytocin on inhibitory synaptic transmission also changes over the course of cortical development.

### Developmental Changes in Oxytocin Receptor Expression in Different Neuronal Types

What biological changes may account for, or at least contribute to, dynamic changes in the effect of oxytocin on synaptic transmission? Oxytocin primarily signals through the oxytocin receptor (OXTR), a G protein-coupled receptor expressed widely in the brain (Gimpl and Fahrenholz, 2001; Jurek and Neumann, 2018). OXTR expression is developmentally dynamic and is regulated by experience (Vaidyanathan and Hammock, 2017). In the mouse cerebral cortex, *Oxtr* mRNA and OXTR protein expression, as well as radioligand labeling of receptors, all showed peak receptor expression at P14 (Hammock and Levitt, 2013; Zheng et al., 2014; Mitre et al., 2016). However, it is not known if *Oxtr* expression is mostly in glutamatergic or GABAergic neurons at this age. We thus performed *in situ* hybridization of *Oxtr* mRNA, in combination with immunohistochemistry for the beta subunit of Ca^2+^/calmodulin-dependent protein kinase II (CaMKIIβ) or somatostatin (SST), respectively labeling glutamatergic (excitatory) neurons or a subclass of GABAergic (inhibitory) neurons previously shown to express *Oxtr* (Nakajima et al., 2014). *Oxtr* *in situ* hybridization was also carried out using *GAD67-GFP* mice, in which GABAergic neurons are labeled with the green fluorescent protein (GFP; Tamamaki et al., 2003).

**Figure 3:**
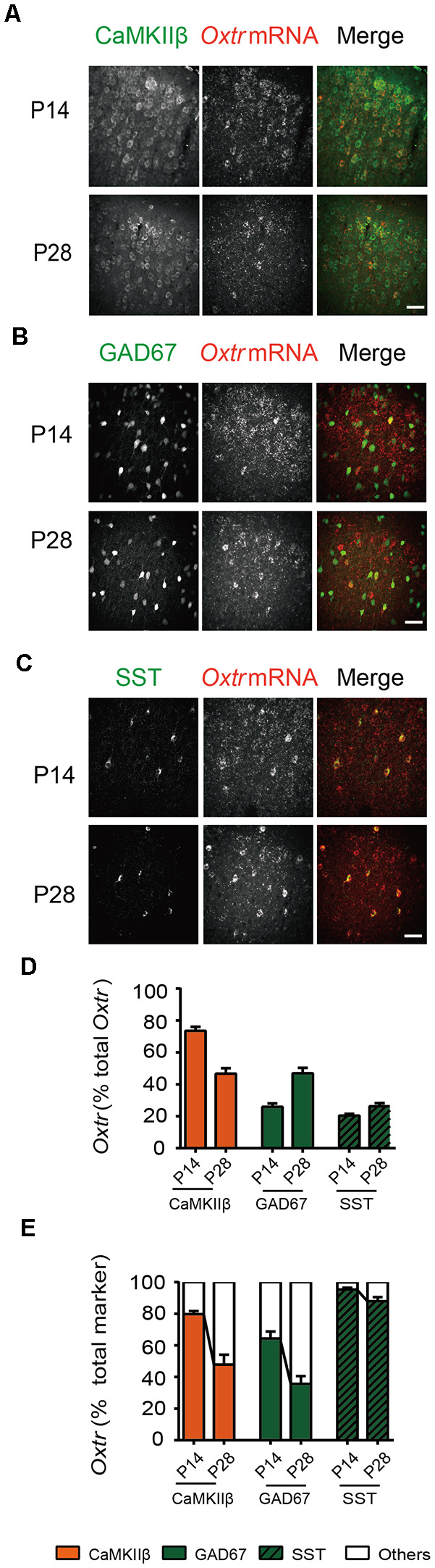
*Oxtr* mRNA expression in glutamatergic and GABAergic neurons. **(A)** Representative images of *Oxtr* mRNA co-labeling with the glutamatergic neuron marker CaMKIIβ, age of mice as indicated in this and subsequent panels. **(B)** Representative images of *Oxtr* mRNA co-labeling with the GABAergic neuron marker GAD67. **(C)** Representative images of *Oxtr* mRNA co-labeling with SST, a marker for a subtype of GABAergic neurons. **(D)** *Oxtr* mRNA expression in different cell types in P14 and P28 mice, normalized to total *Oxtr* expression (P14: CaMKIIβ, 73.56 ± 2.51%; *n* = 22; GAD67, 26.0 ± 2.06%; *n* = 30; SST, 20.45 ± 1.07%;* n* = 30; P28: CaMKIIβ, 46.69 ± 3.42%; *n* = 20; GAD67, 46.99 ± 3.39%; *n* = 28; SST, 26.28 ± 1.98%; *n* = 30). **(E)** *Oxtr* mRNA colocalizing with marker as labeled (CaMKIIβ: P14, 79.79 ± 2.04%; *n* = 22; P28, 47.98 ± 6.17%; *n* = 20; GAD67: P14, 64.39 ± 4.43%; *n* = 30; P28, 35.74 ± 4.86%; *n* = 28; SST: P14, 95.39 ± 1.12% *n* = 30; P28: 87.96 ± 2.66%; *n* = 30). **(A–C)** Scale bar: 50 μm.

In S1, at both P14 and P28, *Oxtr* mRNA partially colocalized with all three markers (Figures 3A–C); colocalization was defined as the overlap between the two signals at the pixel level. Between P14 and P28, the distribution of *Oxtr* mRNA changed, from mostly colocalizing with CaMKIIβ at P14 (CaMKIIβ: 73.56 ± 2.51%; GAD67: 26.0 ± 2.06%; SST: 20.45 ± 1.07%), to relatively even distribution between glutamatergic and GABAergic neurons at P28 (CaMKIIβ: 46.69 ± 3.42%; GAD67: 46.99 ± 3.39%; SST: 26.28 ± 1.98%; Figure 3D). In addition, the ratio of *Oxtr*-expressing cells decreased from P14 to P28 in both glutamatergic neurons (P14: 79.79 ± 2.04%; P28: 47.98 ± 6.17%) and GABAergic neurons (P14: 64.39 ± 4.43%; P28: 35.74 ± 4.86%; Figure 3E), consistent with previous reports showing peak *Oxtr* expression in the cerebral cortex at P14. At both time points, a very high proportion of SST neurons expressed *Oxtr* (P14: 95.39 ± 1.12%; P28: 87.96 ± 2.66%; Figure 3E). These results show that *Oxtr* expression is dynamic during development, and has distinct expression patterns at P14 and P28.

**Figure 4:**
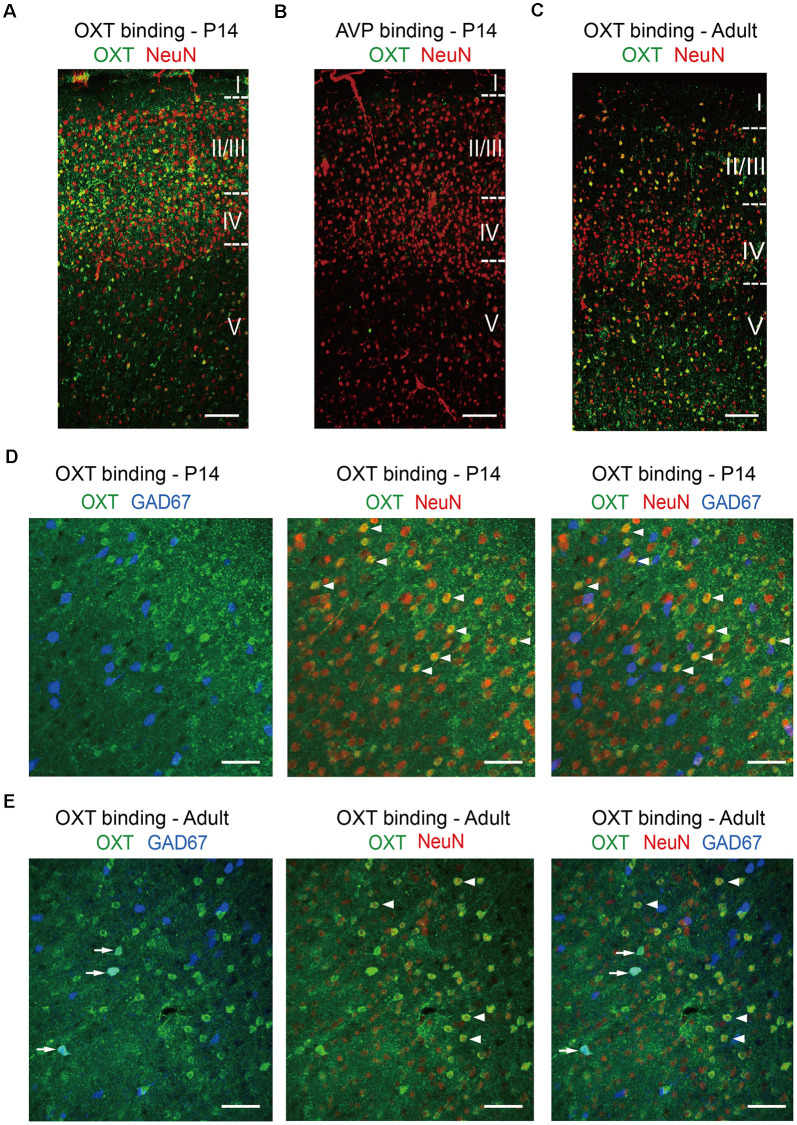
Oxytocin binding in glutamatergic and GABAergic neurons. **(A)** Incubation of P14 acute brain slice with oxytocin leads to significant oxytocin binding and oxytocin antibody labeling (green) in neurons, co-labeled with NeuN (red). **(B)** As a negative control, incubation of P14 acute brain slice with vasopressin does not result in oxytocin antibody labeling. **(C)** Application of oxytocin to an adult brain slice leads to oxytocin antibody labeling (green) with a different pattern, as compared to P14. **(D)** Colocalization of oxytocin (green), NeuN (red), and GAD67 (blue) in the P14 cerebral cortex. **(E)** Colocalization of oxytocin (green), NeuN (red), and GAD67 (blue) in the adult cerebral cortex. Arrows indicate colocalization of oxytocin and GAD67; arrowheads indicated the colocalization of oxytocin and NeuN. **(A–C)** Cortical layers are delineated by dashed lines. Scale bar: 100 μm. **(D,E)** Scale bar: 50 μm.

Not having a specific OXTR antibody on hand, we confirmed our *in situ* results using the “oxytocin binding” method. Persistent activation of OXTR, a G protein-coupled receptor, leads to its endocytosis and internalization, together with its ligand oxytocin (Gimpl and Fahrenholz, 2001); thus cells expressing functional OXTR have significant oxytocin binding capacity and can be labeled using an antibody against oxytocin following ligand binding. In N2a cells, application of oxytocin resulted in specific labeling of OXTR-expressing cells with oxytocin antibody, but not neighboring cells not expressing OXTR; this effect was blocked by pre-incubation with oxytocin antibody and did not occur upon incubation with the closely related neuropeptide vasopressin (Supplementary Figure 2). We then treated acute brain slices with oxytocin for 20 min, fixed the brain slices, and immunostained for oxytocin and the pan-neuronal marker NeuN (Figure 4). In S1 of P14 mice, oxytocin immunoreactivity, marking cells with internalized OXTR, colocalized significantly with NeuN and was relatively high in the superficial layers of the cerebral cortical (layers 2/3), as compared to the deeper layer (layer 5; Figure 4A). As a control for specificity, cortical brain slices incubated with the closely related neuropeptide vasopressin were not labeled with oxytocin antibody (Figure 4B). In S1 of adult mice, the oxytocin immune-reactive cells distributed relatively evenly across superficial and deeper layers (Figure 4C). This dynamic pattern of OXTR expression in different cortical layers during development is consistent with a recent report using an OXTR reporter mouse line (Newmaster et al., 2020). The results of these oxytocin labeling experiments are also consistent with those of our *Oxtr* *in situ* hybridization experiments, although the labeling efficiency is lower. Specifically, the proportion of oxytocin immune-reactive cells that are GAD67-positive increased from 15.31 ± 0.30% at P14 to 29.17 ± 4.17% in adult mice. In both age groups, over 90% (P14: 90.30 ± 3.33%; adult: 90.28 ± 1.39%) of oxytocin immune-reactive cells co-labeled with NeuN, consisting with high OXTR expression in neurons (Figures 4D,E). In addition to the cerebral cortex, internalized oxytocin also colocalized with NeuN in the hippocampus, amygdala, and lateral septum of P14 mice (Supplementary Figures 3A–C). Labeling of cortical neurons was also achieved using FITC-oxytocin (see “Materials and Methods” section for details; Supplementary Figure 4A). Importantly, FITC-oxytocin did not label cells in the PVH of *EIIa-Cre; Oxtr^fl/fl^* (*EIIa-Cre*; *Oxtr* cKO) mice, where *Oxtr* is removed from the very early embryo (Lakso et al., 1996; Lee et al., 2008), thus demonstrating the requirement of OXTR for oxytocin binding *in vivo* (Supplementary Figures 4B,C).

### Altered Excitatory Synaptic Transmission in Oxytocin and OXTR Knockout Mice

**Figure 5:**
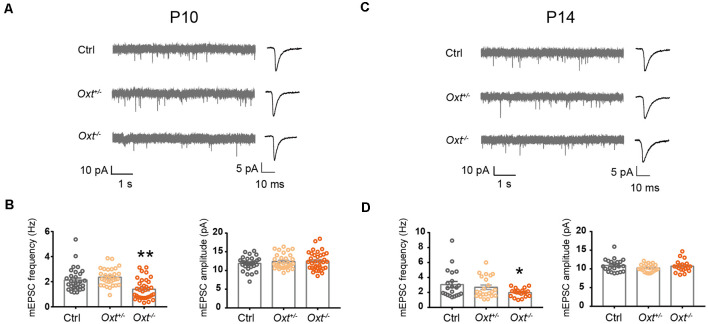
Reduced excitatory synaptic transmission in oxytocin knockout mice. **(A–C)** Representative mEPSC recording (left) and average waveforms (right) of *Oxt^+/−^*, *Oxt^−/−^* mice and littermate Ctrl mice at P10 and P14. **(B)** mEPSC frequency is reduced in P10 *Oxt^−/−^* mice (Ctrl, 2.13 ± 0.17 Hz;* n* = 31; *Oxt^+/−^*, 2.36 ± 0.12 Hz; *n* = 31; *Oxt^−/−^*, 1.40 ± 0.14 Hz; *n* = 35; *P* < 0.01 vs. Ctrl), while mEPSC amplitude is not significantly affected (Ctrl, 11.88 ± 0.32 pA; *Oxt^+/−^*, 12.39 ± 0.35 pA; *Oxt^−/−^*, 12.48 ± 0.41 pA). **(D)** mEPSC frequency is reduced in P14 *Oxt^−/−^* mice (Ctrl, 3.06 ± 0.42 Hz; *n* = 21; *Oxt^+/−^*, 2.69 ± 0.30 Hz; *n* = 22; *Oxt^−/−^*, 1.98 ± 0.12 Hz; *n* = 20; *P* < 0.05 vs. Ctrl), while mEPSC amplitude is not significantly affected (Ctrl, 10.95 ± 0.38 pA; *Oxt^+/−^*, 10.25 ± 0.22 pA; *Oxt^−/−^*, 10.74 ± 0.35 pA). One-way ANOVA followed by Tukey’s multiple comparison test for all panels. *P < 0.05, **P < 0.01, unpaired *t*-test.

Having shown the sufficiency of oxytocin to regulating excitatory synaptic transmission, as well as expression of OXTR in a large proportion of L2/3 pyramidal neurons at P14, we next asked if oxytocin and OXTR are required for regulation of excitatory synaptic transmission under basal conditions and/or following oxytocin application. In homozygous oxytocin knockout mice (homo, *Oxt^−/−^*, Young et al., 1996), mEPSC frequency was significantly reduced, as compared with littermate wildtype (WT, *Oxt^+/+^*) or heterozygous (het, *Oxt^+/−^*) mice, at both P10 (WT: 2.13 ± 0.17 Hz, het: 2.36 ± 0.12 Hz, homo: 1.40 ± 0.14 Hz; WT vs. het, *P* = 0.52; WT vs. homo *P* < 0.01) and P14 (WT: 3.06 ± 0.42 Hz, het: 2.69 ± 0.30 Hz, homo: 1.98 ± 0.12 Hz; WT vs. het: *P* = 0.68; WT vs. homo: *P* < 0.05; Figure 5). mEPSC frequency of heterozygous mice was not significantly different from wildtype littermates at both ages, and mEPSC amplitude was not significantly different between all genotypes at both ages (Figure 5). Thus, oxytocin’s loss-of-function affects excitatory synaptic transmission as early as P10.

**Figure 6:**
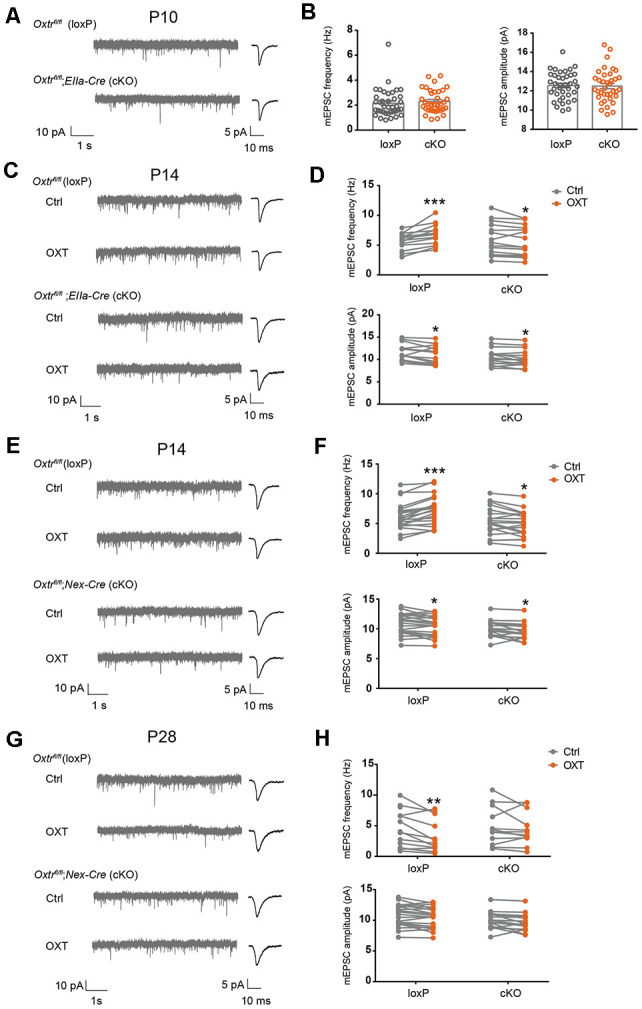
Blockade of oxytocin-induced changes in excitatory synaptic transmission in *Oxtr*cKO mice. **(A)** Representative mEPSC recording (left) and average waveforms (right) of P10 *EIIa-Cre;Oxtr* cKO mice and littermate loxP controls. **(B)** mEPSC frequency (Ctrl, 2.16 ± 0.18 Hz, *n* = 38; cKO, 2.30 ± 0.15 Hz, *n* = 37; *P* = 0.54, unpaired *t-*test) and amplitude (Ctrl: 12.55 ± 0.23 pA; cKO, 12.50 ± 0.28 pA; *P* = 0.90, unpaired *t*-test) were similar between *EIIa-Cre;Oxtr* cKO mice and loxP littermates. **(C,E,G)** Representative mEPSC recordings (left) and average waveforms (right) before (Ctrl) and after (OXT) oxytocin application, age and genotype as indicated. **(D)** Oxytocin application increased mEPSC frequency but reduced mEPSC amplitude in P14 control mice, while its application reduced both mEPSC frequency and amplitude in P14 *EIIa-Cre;Oxtr* cKO mice (frequency: loxP, Ctrl, 5.69 ± 0.27 Hz, OXT, 6.78 ± 0.33 Hz, *n* = 14; *P* < 0.001, paired *t*-test; cKO, Ctrl, 5.91 ± 0.48 Hz, OXT, 5.56 ± 0.45 Hz, *n* = 16; *P* < 0.05, paired *t*-test; amplitude: loxP, Ctrl, 11.61 ± 0.37 pA, OXT, 11.0 ± 0.41 pA; *P* < 0.05, paired *t*-test; cKO: Ctrl, 10.58 ± 0.34 pA, OXT, 10.24 ± 0.34 pA; *P* < 0.05, paired *t*-test). **(F)** Oxytocin application increased mEPSC frequency, but reduced mEPSC amplitude in P14 loxP mice, while its application reduced both mEPSC frequency and amplitude in *Nex-Cre;Oxtr* cKO mice (frequency: loxP, Ctrl, 6.22 ± 0.49 Hz, OXT, 7.25 ± 0.53 Hz, *n* = 21; *P* < 0.001, paired *t*-test; cKO: Ctrl, 5.44 ± 0.55 Hz, OXT, 4.98 ± 0.50 Hz, *n* = 19; *P* < 0.05, paired *t*-test; amplitude: loxP, Ctrl, 10.79 ± 0.39 pA, OXT, 10.43 ± 0.37 pA; *P* < 0.05, paired *t*-test; cKO: Ctrl, 9.99 ± 0.31 pA, OXT, 9.56 ± 0.31 pA; *P* < 0.05, paired *t*-test). **(H)** Oxytocin application reduced mEPSC frequency, without affecting mEPSC amplitude in P28 loxP mice; its application did not affect mEPSC frequency or amplitude in *Nex-Cre;Oxtr* cKO mice (frequency: loxP, Ctrl, 4.49 ± 0.81 Hz, OXT, 3.30 ± 0.70 Hz, *n* = 14; *P* < 0.01, paired *t*-test; cKO: Ctrl, 4.92 ± 0.89 Hz, OXT, 4.49 ± 0.78 Hz, *n* = 12; *P* = 0.35, paired *t*-test; amplitude: loxP, Ctrl, 8.12 ± 0.39 pA, OXT, 7.96 ± 0.47 pA; *P* = 0.48, paired *t*-test; cKO: Ctrl, 8.62 ± 0.37 pA, OXT, 8.53 ± 0.36 pA; *P* = 0.72, paired *t-*test). *P < 0.05, **P < 0.01, ***P < 0.001.

In complementary experiments, we examined excitatory synaptic transmission in *EIIa-Cre;Oxtr^fl/fl^* (*EIIa-Cre*;*Oxtr* cKO) mice. *Oxtr* mRNA level was significantly reduced in cortex, hippocampus, and hypothalamus of *EIIa-Cre;Oxtr* cKO mice (Supplementary Figure 4D). At P10, mEPSC frequency (loxP: 2.16 ± 0.18 Hz, cKO: 2.30 ± 0.15 Hz, *P* = 0.54) and amplitude (loxP: 12.55 ± 0.23 pA, cKO: 12.50 ± 0.28 pA, *P* = 0.90) were not significantly different between *EIIa-Cre*;*Oxtr* cKO mice, and *Oxtr^fl/fl^* (loxP) littermates (Figures 6A,B). At P14, while the baseline mEPSC frequency (*P* = 0.78) and amplitude (*P* = 0.15) were not different between the two groups, oxytocin perfusion onto acute brain slices from *EIIa-Cre*;*Oxtr* cKO mice led to a significant reduction in mEPSC frequency (cKO Ctrl: 5.91 ± 0.48 Hz, cKO OXT: 5.56 ± 0.45 Hz, *P* < 0.05), as compared to a significant increase observed in loxP littermates (loxP Ctrl: 5.69 ± 0.27 Hz, loxP OXT: 6.78 ± 0.33 Hz, *P* < 0.001; Figures 6C,D) and in wildtype mice (Figures 1A,B). In both conditions, mEPSC amplitude was reduced following oxytocin application (loxP Ctrl: 11.61 ± 0.37 pA, loxP OXT: 11.0 ± 0.41 pA, *P* < 0.05; cKO Ctrl: 10.58 ± 0.34 pA, cKO OXT: 10.24 ± 0.34 pA, *P* < 0.05; Figures 6C,D).

We further confirmed these results using *Nex-Cre; Oxtr^fl/fl^* (*Nex-Cre; Oxtr* cKO) mice, where Cre is expressed in all excitatory neurons of the cerebral cortex and hippocampus from the late embryo (Goebbels et al., 2006). In P14 *Nex-Cre; Oxtr* cKO mice, oxytocin application also reduced mEPSC frequency (cKO Ctrl: 5.44 ± 0.55 Hz, cKO OXT: 4.98 ± 0.50 Hz, *P* < 0.05) and amplitude (cKO Ctrl: 9.99 ± 0.31 pA, cKO OXT: 9.56 ± 0.31 pA, *P* < 0.05), while the same treatment increased mEPSC frequency (loxP Ctrl: 6.22 ± 0.49 Hz, loxP OXT: 7.25 ± 0.53 Hz, *P* < 0.001) and reduced mEPSC amplitude (loxP Ctrl: 10.79 ± 0.39 pA, loxP OXT: 10.43 ± 0.37, pA, *P* < 0.05) in loxP littermates (Figures 6E,F). Baseline mEPSC frequency (*P* = 0.29) and amplitude (*P* = 0.12) were not significantly different between the two genotypes. Since *Nex-Cre* is only expressed in glutamatergic neurons, these results further confirm requirement for *Oxtr* expression in glutamatergic neurons for oxytocin-dependent regulation of excitatory synaptic transmission.

Measuring excitatory synaptic transmission in* Nex-Cre; Oxtr* cKO mice at P28, we found that baseline mEPSC frequency (*P* = 0.72) and amplitude (*P* = 0.36) were not significantly different between loxP and cKO groups. Oxytocin application to loxP mice reduced mEPSC frequency (loxP Ctrl: 4.49 ± 0.81 Hz, loxP OXT: 3.30 ± 0.70 Hz, *P* < 0.01; Figures 6G,H), similar to its effects on wildtype mice at this age (Figures 1E,F). In contrast, oxytocin application in *Nex-Cre; Oxtr* cKO did not significantly affect mEPSC frequency (cKO Ctrl: 4.92 ± 0.89 Hz, cKO OXT: 4.49 ± 0.78 Hz, *P* = 0.35; Figures 6G,H). In both genotypes, mEPSC amplitude was not affected by oxytocin application (loxP Ctrl: 8.12 ± 0.39 pA, loxP OXT: 7.96 ± 0.47, pA, *P* = 0.48; cKO Ctrl: 8.62 ± 0.37 pA, cKO OXT: 8.53 ± 0.36 pA, *P* = 0.72; Figures 6G,H). Together, these results show that while conditional knockout of *Oxtr* does not affect baseline excitatory synaptic transmission, it inhibits the effect of acute oxytocin application onto brain slices, at both P14 and P28.

## Discussion

### Oxytocin and Sensory Experience Have Similar Sensitive/Critical Periods in Regulating Excitatory Synaptic Transmission of L2/3 Neurons

Here, we found that sensory experience and oxytocin regulate excitatory synaptic transmission in L2/3 pyramidal neurons of the sensory cortices with a similar sensitive/critical period, peaking around P14 and ending around P18 (Figure 1 and Supplementary Figures 1A–D; Zheng et al., 2014). Consistently, sensory experience regulates oxytocin expression with a similar time course, elevation at P14, and essentially no changes at P18 (Supplementary Figures 1E,F; Zheng et al., 2014). Curiously, we observed an increase in oxytocin level in S1 of DR mice (Supplementary Figure 1F), possibly due to homeostatic compensation. The effects of sensory experience and oxytocin on excitatory synaptic transmission are both directional, with sensory deprivation and loss-of-function of oxytocin reducing excitatory synaptic transmission, and environmental enrichment and exogenous oxytocin application increasing synaptic transmission (Figures 1, 5 and Supplementary Figure 1; Zheng et al., 2014). The above evidence suggests that oxytocin may function as a mediator of early experience-dependent plasticity in L2/3 pyramidal neurons of the sensory cortices.

As to what biological change ends the sensitive/critical period, we can only speculate. Oxytocin expression in the hypothalamus increases steadily between P7 and P60 (Zheng et al., 2014), thus it is unlikely that a sharp change in oxytocin expression leads to closure of this sensitive/critical period. *Oxtr* mRNA, OXTR protein, oxytocin binding capacity, and OXTR reporter expression in the sensory cortices has been reported to peak around P14 and drops significantly at P21 and P28 (Hammock and Levitt, 2013; Mitre et al., 2016; Newmaster et al., 2020; Figure 3). Thus, a reduction in *Oxtr* expression may contribute to the closure of the sensitive/critical period. It is probably one of many factors that contribute. To better understand this form of experience-dependent plasticity in the sensory cortices, more mechanistic studies, as well as a deeper understanding of the physiological function of this sensitive/critical period is needed.

### Effects of Oxytocin on Synaptic Transmission at P14

At P14 (and the earlier time point of P10), loss-of-function of oxytocin reduces mEPSC frequency of S1BF L2/3 pyramidal neurons, while its application to acute brain slices increases mEPSC frequency (Figures 1, 5). Furthermore, loss-of-function of *Oxtr* blocks oxytocin-induced increase in mEPSC frequency (Figure 6). *Oxtr* expression is high in L2/3 glutamatergic neurons and low in GABAergic neurons at this age (Figures 3, 4). Consistently, oxytocin application does not significantly affect inhibitory synaptic transmission. Increased excitatory synaptic transmission and no change in inhibition add to an increase in total excitatory input of L2/3 pyramidal neurons.

An interesting fine point is the difference between the effect of *Oxt^−/−^* knockout and *Oxtr* cKO: *Oxt^−/−^* mice have reduced excitatory synaptic transmission under baseline conditions, at both P10 and P14 (Figure 5), while in *EIIa-Cre; Oxtr* cKO or *Nex-Cre; Oxtr* cKO mice, baseline excitatory synaptic transmission is unaffected, but the effect of oxytocin application is blocked (Figure 6). Given that *EIIa-Cre* is expressed from the early embryo, before implantation (Lakso et al., 1996), *EIIa-Cre; Oxtr* cKO mice should have completely or near complete knockout of *Oxtr*. This opens up the possibility that oxytocin may have developmental effects independent of its receptors.

Another subject of interest is whether oxytocin affects mEPSC amplitude, a parameter that correlates with the size of individual synapses. In *Oxt^−/−^* mice, and in *Oxtr* cKO mice, mEPSC amplitude was similar between knockout mice and littermate controls (Figures 5, 6). Thus loss-of-function of *Oxt* or *Oxtr* does not affect mEPSC amplitude. In all oxytocin application experiments, however, a small but significant reduction in mEPSC amplitude was often observed (Figures 1, 6). A small reduction in mEPSC amplitude, in addition to being a biological phenomenon, could also be an artifact due to increased serial resistance during the course of whole cell patch-clamp recordings. To minimize this problem, we only analyzed recordings in which series and input resistances changed by less than 20% over the course of the experiment. Also, in P18 mice, neither mEPSC amplitude nor frequency was affected by oxytocin application (Figures 1C,D). Thus, the small reduction in mEPSC amplitude following oxytocin application is likely to be a *bona fide* biological phenomenon. However, since the magnitude of the increase in mEPSC frequency is much larger than the reduction in mEPSC amplitude, the total charge transfer, representing total excitatory input of the neuron, is increased following oxytocin application at P14.

### Effects of Oxytocin on Synaptic Transmission at P28

In P28 mice, oxytocin application reduces mEPSC frequency and increases mIPSC frequency (Figures 1, 2) of S1BF L2/3 pyramidal neurons. The effect of acute oxytocin application on reducing mEPSC frequency was confirmed by *in vivo* oxytocin injection (Figures 1H–J) and blocked in *Nex-Cre; Oxtr* cKO mice (Figures 6G,H).

These results, together with those of P14, suggest that oxytocin has distinct effects on synaptic transmission at P14 and P28: increasing total excitatory inputs (increased mEPSC frequency and no changes in inhibition) of L2/3 pyramidal neurons at P14, and reducing it (reduced mEPSC frequency and increased mIPSC frequency) at P28. What physiological changes may underlie these switches? As discussed above, oxytocin expression increases steadily between P7 and P60 (Zheng et al., 2014), and thus is unlikely to account for the above described switch. Our *in situ* hybridization and oxytocin binding results, together with published data using a variety of approaches to measure OXTR level (Hammock and Levitt, 2013; Mitre et al., 2016; Newmaster et al., 2020), suggest that OXTR level is higher at P14, as compared to P21 and P28. It is relatively straightforward for high receptor expression to be associated with a higher level of signal transduction and increased transmission (e.g., P10 and P14), and lower receptor expression to be associated with no changes (e.g., P18); however, the change from increase at P14 to reduction at P28 presumably requires additional alterations in downstream signal transduction components.

In terms of GABAergic synaptic transmission, our results (Figures 3, 4) suggest increased relative expression of OXTR in GABAergic neurons in P28 and older mice, as compared to P14. An increase in OXTR expression in GABAergic cells would presumably increase OXTR-dependent signaling in these cells. Since we observed an increase in GABAergic input to L2/3 pyramidal neurons at P28, the increased OXTR expression likely enhanced the synaptic output of GABAergic neurons onto pyramidal neurons. A recent study indeed showed that oxytocin can enhance the excitability of SST neurons, thereby reducing the level of spontaneous activity (Maldonado et al., 2021).

Thus, combining the results on glutamatergic and GABAergic transmission, it seems that lower expression of OXTR is associated with no effects on synaptic transmission, while higher OXTR expression is associated with increased transmission. The exception is excitatory synaptic transmission in P28 and older mice, where oxytocin application significantly reduces excitatory synaptic transmission in neurons expressing a low level of OXTR. We hypothesize that change in the level of one or more OXTR downstream signaling component mediates this effect.

We focused in S1 L2/3 pyramidal neurons for this study. Recent work showed that oxytocin affects spontaneous network events differentially in S1 and V1 (Maldonado et al., 2021). Given the complexity of the effects of oxytocin, studies on more cell types, more brain regions, and at more developmental stages, as well as more in-depth investigations of OXTR downstream signaling under these different conditions, are needed for a full understanding of its function.

### Implications for Neural Circuit Development and Plasticity

Oxytocin has been shown to affect many aspects of neural circuit development and function, including regulating excitatory or inhibitory synaptic transmission, altering neuronal firing rates and patterns, and modulating the transition of GABA from excitatory to inhibitory (Stoop, 2012; Hammock, 2015; Marlin and Froemke, 2017; Ben-Ari, 2018). Our study adds to existing knowledge by showing that oxytocin regulates the excitatory and inhibitory synaptic transmission of L2/3 pyramidal neurons in a developmentally dynamic manner. An immediate implication of this finding is that giving the same dose of oxytocin to an individual may have different effects, sometimes opposite, depending on the developmental stage of the individual.

Because oxytocin can promote trust, eye contact, and facial memory, it has been proposed as a therapy for the treatment of autism spectrum disorders (ASD), a developmental disorder with deficits in social communication (Green and Hollander, 2010; Insel, 2010; Meyer-Lindenberg et al., 2011; Yamasue et al., 2012; Miller, 2013). A very large proportion of individuals with ASD also are hypo- or hypersensitive to sensory inputs (Marco et al., 2011; Suarez, 2012), consistent with the function of oxytocin in regulating cortical neural circuit wiring. However, clinical trials investigating the effectiveness of oxytocin as a treatment for ASD reported mixed results (Guastella and Hickie, 2016; Ooi et al., 2017; Keech et al., 2018; Huang et al., 2021). The dynamic developmental effects of oxytocin likely add to the difficulty of obtaining consistent results. Given the heterogeneity of ASD, the developmental switch point at which oxytocin function shifts from being overall excitatory in L2/3 pyramidal neurons to overall inhibitory may be different for different individuals. If we consider that oxytocin regulates the function of many types of neurons and that many of these functions may be developmentally dynamic and brain-region specific, the situation becomes exceedingly complex.

In addition to identifying complexity, what potential directions do we see moving forward? First, a deeper understanding of oxytocin-OXTR downstream signaling, as well as of other pathways mediating oxytocin signaling, would be important to understand the diversity of its physiological functions. Second, taking developmental stage/age into account may contribute towards more consistent results in both animal and human studies. The developmental stage/age may need to be defined functionally, and therapies may have to optimize treatment windows. Third, especially for patient studies, attempts to subtype or subclass may reduce the heterogeneity of outcomes. In the end, we hope that deeper mechanistic insights eventually translate to effective therapies for patients.

## Data Availability Statement

The raw data supporting the conclusions of this article will be made available by the authors, without undue reservation.

## Ethics Statement

The animal study was reviewed and approved by Institutional Animal Care and Use Committee at the Institute of Neuroscience, Chinese Academy of Sciences and of Peking University.

## Author Contributions

JZ, S-JL, WM, XZ, and J-JZ performed experiments. JZ and XY wrote the manuscript. CW designed and provided reagents. All authors designed experiments, revised and approved the manuscript. All authors contributed to the article and approved the submitted version.

## Conflict of Interest

The authors declare that the research was conducted in the absence of any commercial or financial relationships that could be construed as a potential conflict of interest.
