# Supplementary material for: Oxytocin Regulates Synaptic Transmission in the Sensory Cortices in a Developmentally Dynamic Manner
Source: Front Cell Neurosci. 2021 Jun 9;15:673439. doi: 10.3389/fncel.2021.673439 (PMC8221398; doi:10.3389/fncel.2021.673439)
Supplement: Supplementary file 1 [file Presentation_1.pdf]

Supplementary Material

Oxytocin regulates synaptic transmission in the sensory cortices in a developmentally dynamic manner

Jing Zhang[1,2,†,*], Shu-Jing Li[1,†], Wanying Miao[1,†], Xiaodi Zhang[1,†], Jing-Jing Zheng[1], Chen Wang[3] and Xiang Yu,[1,4, *]

[1]Institute of Neuroscience, State Key Laboratory of Neuroscience, Center for Excellence in Brain Science and Intelligence Technology, Chinese Academy of Sciences, Shanghai 200031, China

[2]Department of Clinical Pharmacology, Pharmacy College, Nanjing Medical University, Nanjing, China

[3]CAS Key Laboratory of Biological Effects of Nanomaterials and Nanosafety, CAS Center for Excellence in Nanoscience, National Center for Nanoscience and Technology, Beijing 100190, P. R. China

[4]School of Life Sciences, Peking-Tsinghua Center for Life Sciences, and Peking University McGovern Institute, Peking University, Beijing 100871, China

[†]These authors share first authorship

[*]Correspondence to: zj1984@njmu.edu.cn, or yuxiang01@pku.edu.cn

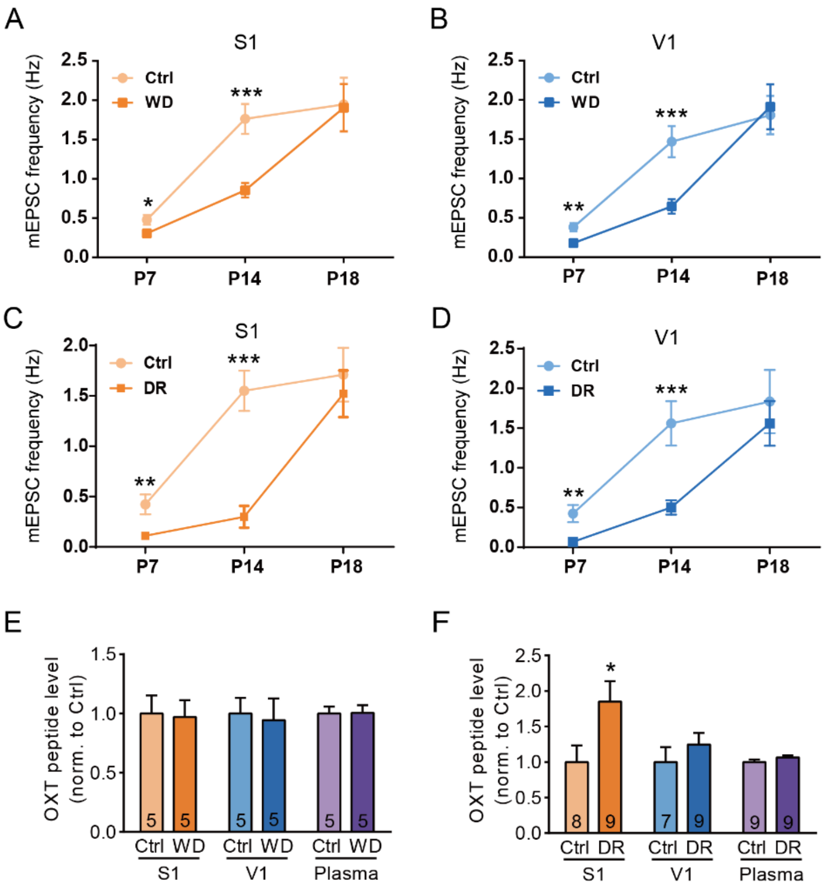

Supplementary Figure S1. The effect of sensory deprivation on excitatory synaptic transmission in S1BF and V1, and on oxytocin expression. (A) Developmental changes in mEPSC frequency in S1BF of Ctrl and WD mice at P7 (replotted from data presented in Supplementary Figure 1G of Zheng *et al.*, 2014), P14 (replotted from data presented in Figure 1B of Zheng *et al.*, 2014) and P18 (P18, Ctrl, 1.95 ± 0.34 Hz, n = 13; WD, 1.91 ± 0.30 Hz, n = 14; P = 0.93). (B) Developmental changes in mEPSC frequency in V1 of Ctrl and WD mice at P7 (replotted from data presented in Supplementary Figure 1H of Zheng *et al.*, 2014), P14 (replotted from data presented in Figure 1G of Zheng *et al.*, 2014) and P18 (P18, Ctrl, 1.81 ± 0.25 Hz, n = 11; WD, 1.91 ± 0.29 Hz, n = 12; P = 0.78). (C) Developmental changes in mEPSC frequency in S1BF of Ctrl and DR mice at P7 (replotted from data presented in Supplementary Figure 1I of Zheng *et al.*, 2014), P14 (replotted from data presented in Figure 3B of Zheng *et al.*, 2014) and P18 (Ctrl, 1.71 ± 0.27 Hz, n = 9; DR, 1.52 ± 0.23 Hz, n = 18; P = 0.62). (D) Developmental changes in mEPSC frequency in V1 of Ctrl and DR mice at P7 (replotted from data presented in Supplementary Figure 1J of Zheng *et al.*, 2014), P14 (replotted from data presented in Figure 3B of Zheng *et al.*, 2014) and P18 (Ctrl, 1.83 ± 0.40 Hz, n = 11; DR, 1.56 ± 0.28 Hz, n = 17; P = 0.57). (E) Oxytocin peptide level in S1, V1 and plasma of P18-P21 mice, normalized to Ctrl (S1: Ctrl, 1 ± 0.15; WD, 0.97 ± 0.14; P = 0.89; V1: Ctrl, 1 ± 0.13; WD, 0.94 ± 0.18; P = 0.81; Plasma: Ctrl, 1 ± 0.06; WD, 1.00 ± 0.07; P = 0.96). (F) Oxytocin peptide level in S1, V1 and plasma of P18-P21 Ctrl and DR mice (S1: Ctrl, 1 ± 0.24; DR, 1.85 ± 0.28; P = 0.04; V1: Ctrl, 1 ± 0.21; DR, 1.25 ± 0.17; P = 0.36; Plasma: Ctrl, 1 ± 0.04; DR, 1.07 ± 0.03; P = 0.17). Unpaired two-tailed Student's *t*-tests were used for all comparisons.

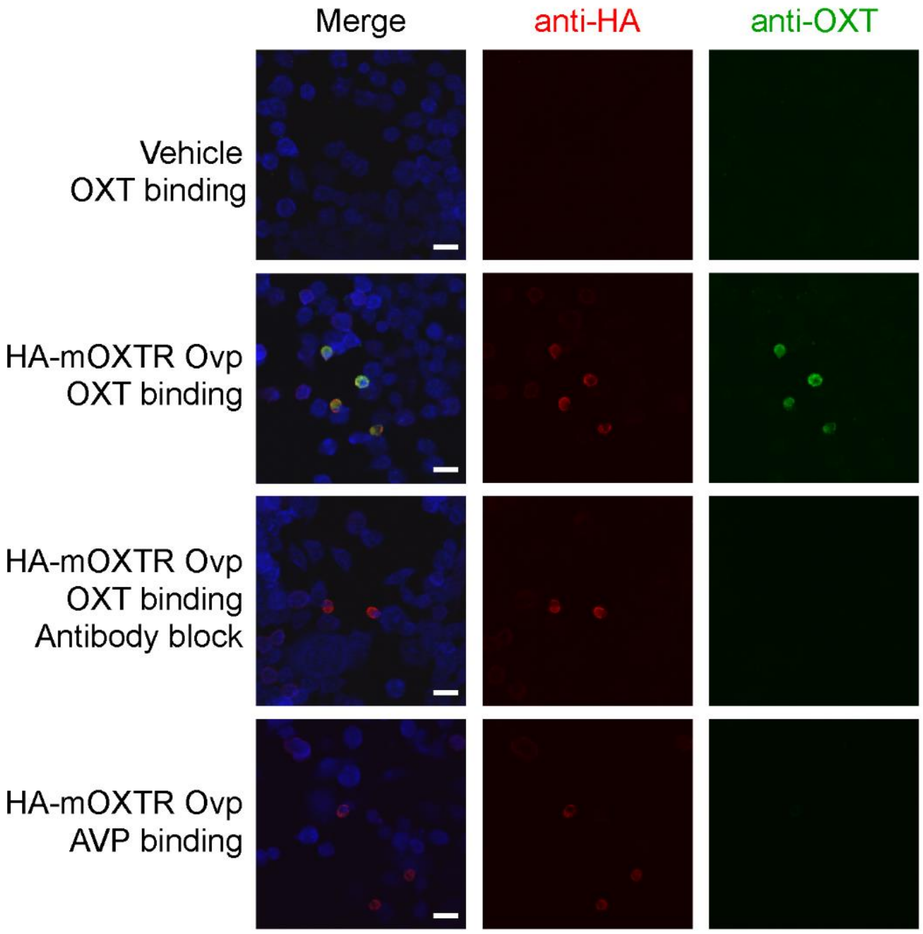

Supplementary Figure S2. Specificity of oxytocin binding to OXTR in N2a cells. Representative images of N2a cells immunostained for oxytocin (anti-OXT, green), HA-tag (anti-HA, red) and counterstained with Nissl (blue) after different conditions. Row 1 (Vehicle + OXT binding), cells transfected with empty vector but subjected to oxytocin binding experiment; Row 2 (HA-mOXTR Ovp + OXT binding), cells transfected with HA-mOXTR-expressing vector and subjected to oxytocin binding experiment; Row 3 (HA-mOXTR Ovp + OXT binding + Antibody block), same as row 2 but anti-OXT antibody was pre-incubated with excessive oxytocin peptide; Row 4 (HA-mOXTR Ovp + AVP binding), same as row 2 except vasopressin peptide was used. Scale bars, 20 μm.

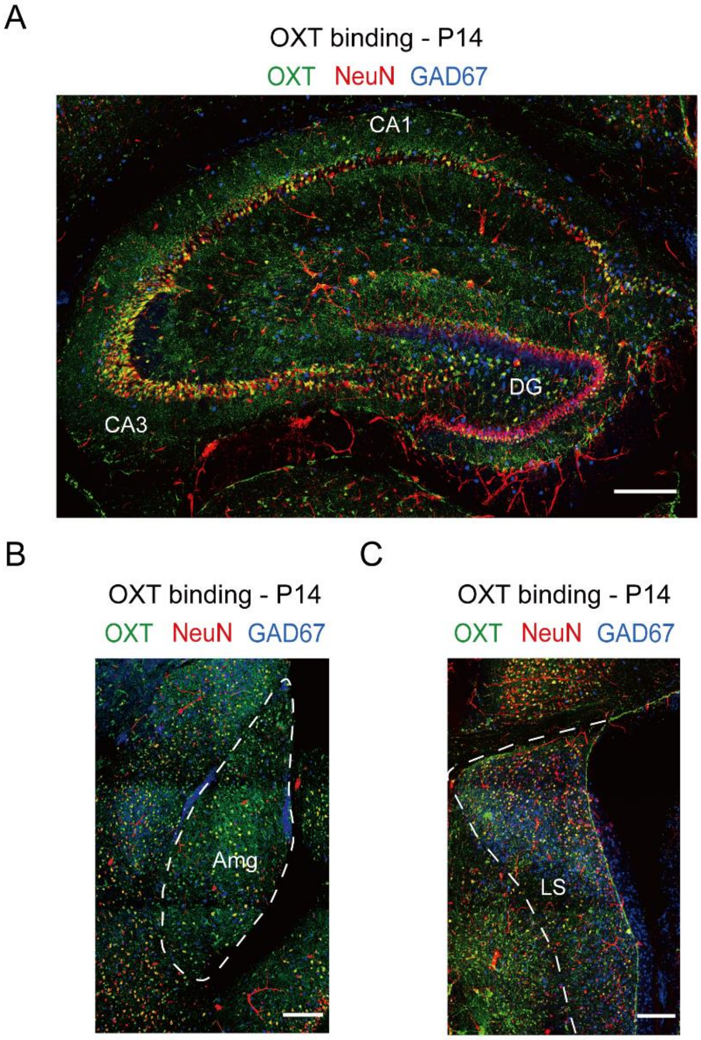

Supplementary Figure S3. Oxytocin binding in different brain regions in P14 mice. (A-C) Incubation of P14 acute brain slice with oxytocin leads to significant oxytocin binding and oxytocin antibody labelling (green) in the hippocampus (A), amygdala (B) and lateral septum (LS) (C), colabelling with both NeuN (red) and GAD67 (blue). Scale bar: 200 μm.

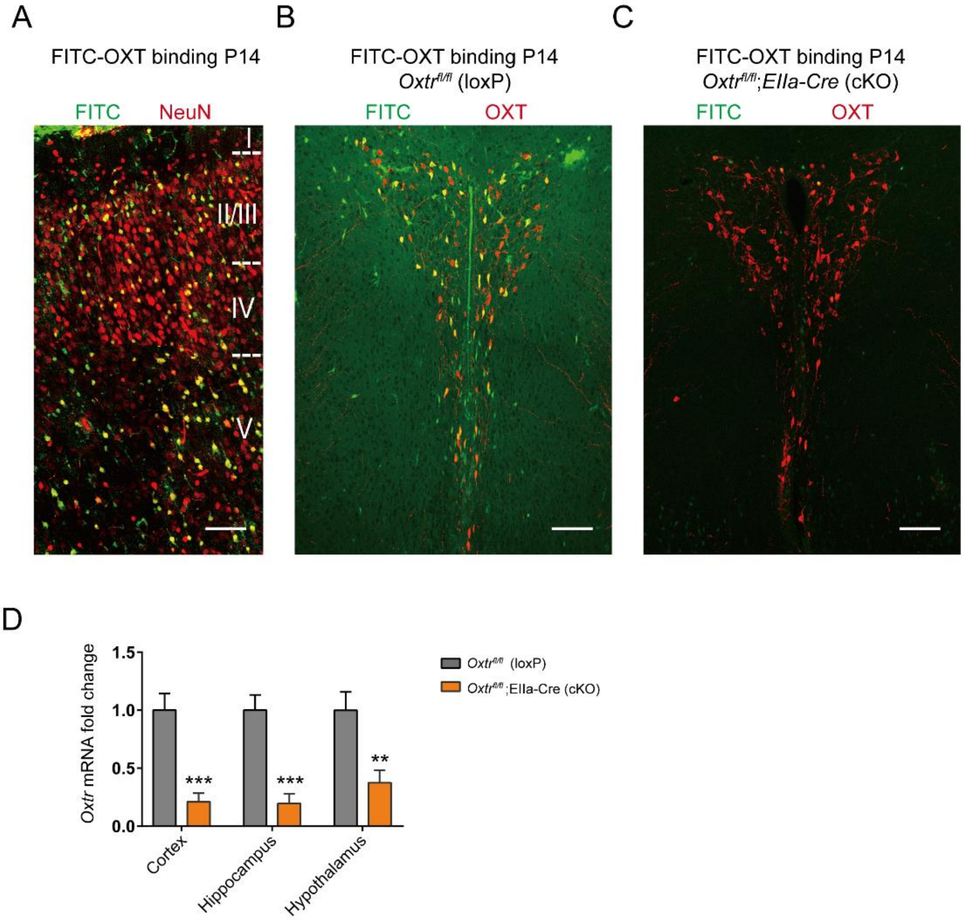

Supplementary Figure S4. Oxytocin binding requires OXTR expression. (A) Incubation of acute slices of P14 cerebral cortex with FITC-oxytocin led to significant FITC-oxytocin labelling (green) in neurons (labelled with NeuN, red). Cortical layers are delineated by dashed lines. (B) *In vivo s*tereotaxic injection of FITC-oxytocin (1 µM, 1 µl, unilateral, green) resulted in significant colocalization with oxytocin immunostaining (red) in P14 *Oxtr*$^{fl/fl}$ (loxP) mice. (C) The same injection in *Oxtr*$^{fl/fl}$*; EIIa-Cre* (cKO) mice led to almost no FITC-oxytocin labelling, demonstrating requirement of OXTR expression for FITC-oxytocin labelling. (A-C) Scale bar: 100 µm, (D) The expression of *Oxtr* mRNA in different brain region of *Ella-Cre;Oxtr* cKO and littermate loxP mice (cortex: Ctrl, 1 ± 0.14; cKO, 0.20 ± 0.08; $n = 6$; P < 0.001, unpaired *t*-test; hippocampus: Ctrl, 1 ± 0.13; cKO, 0.19 ± 0.08; $n = 6$; $P < 0.001$, unpaired *t*-test; hypothalamus: Ctrl, 1 ± 0.16; cKO, 0.37 ± 0.11; $n = 6$; $P < 0.01$, unpaired *t*-test).
